# Assessing the Linkage between Exposure to Violence and Victimization, Coping, and Adjustment among Urban Youth: Findings from a Research Study on Adolescents

**DOI:** 10.3390/children6030036

**Published:** 2019-02-27

**Authors:** Zina McGee, Chelsea Alexander, Khasya Cunningham, Celine Hamilton, Courtney James

**Affiliations:** Department of Sociology, Hampton University, Hampton, VA 23668, USA; chelsea.alexander@my.hamptonu.edu (C.A.); khasya.cunningham@my.hamptonu.edu (K.C.); celine.hamilton@my.hamptonu.edu (C.H.); courtney.james1@my.hamptonu.edu (C.J.)

**Keywords:** coping, peer violence, victimization, adjustment, delinquency, anxiety, depression

## Abstract

From examinations of the literature on the influence that exposure to violence and coping strategies have on delinquent behavior and emotional outcomes, this study addresses the association between violent victimization and the moderating effects of coping strategies among 500 African-American adolescents who exhibit both externalizing behaviors such as delinquency and internalizing symptoms, including anxiety and depression. The investigation examines the development of the aforementioned adjustment problems in response to victimization, and the findings indicate a relationship between the specific indices of victimization, including peer violence, and the symptomatology and coping mechanisms utilized by the youth in this study. Suggestions for future research in this area are discussed.

## 1. Introduction

A review of the literature suggests that violent victimization in communities and schools remains a stressor paramount among children and adolescents, gravely influencing their healthy developmental outcomes. Several research studies have explored the relationship between victimization, including peer violence, and both behavioral consequences and emotional adjustment. Results have suggested that many youths will exhibit subsequent internalizing symptoms and disruptive behavior in response to patterns of violent events [1,2,3]. With regard to exposure to violence, many youths are the primary witnesses to criminal activity in disadvantaged settings, while peer violence and other forms of direct victimization remain prevalent primarily among those between the ages of 12–18 [4]. Even more disturbing is that a substantial proportion of crimes within urban communities and schools are committed by African-American youth against other African-American youth, forcing many of them to respond to their victimization status with repeated delinquency and ensuing violence [4]. While it is widely known that African-American youths in these violent communities are at an increased risk for both internalizing and externalizing symptomatology, less is revealed about the level at which coping may serve as a buffer or mitigate the effects of levels of victimization in particular. Studies that have pointed toward the impact of direct and indirect victimization tend to include specific types of victimization as indicators of direct exposure, while fewer analyses have examined variations in coping strategies among the rates and type of violent victimization as part of a composite variable [5,6,7]. Moreover, research has shown that the coping strategies employed by minority youths can either increase or decrease their likelihood of engaging in delinquent behavior, and this relationship can be impacted by gender, age, and family structure. More specifically, the cultural variance model highlights the differences in environmental impacts that result from adaptive actions, social outlooks, and emotional functioning [8]. The model assumes that minority adolescents are resistant, competent, and capable of problem solving in stress-inducing situations. Hence, the context and meanings within the dominant culture cannot be used to explain the particular behavior of minority youth, as the model purports that family and community functions can produce culturally distinctive structures and dynamics, including those relating to coping and adjustment among members of this population.

As emotional adjustment and externalizing symptoms continue to impact juveniles exposed to violence in various contexts, research studies have shown that coping patterns may differ across levels of victimization, particularly victimization by peers. For example, one study examined the profiles of internalizing and externalizing symptoms that accompany bullying behavior and bullying victimization [9]. The internalizing symptoms involved anxiety and depression, while the externalizing symptoms included delinquency and violence against peers. The results showed that there was little evidence on the differentiating effects on indirect, direct, and dual victimization on symptom profiles. Evidence also showed that those who were categorized as high internalizing and high externalizing were more likely to be in this category if they had been exposed to any type of bullying, prompting the authors to note that the frequency of bullying may have an effect on the development of internalizing symptoms. Other research has examined coping strategies as potential moderators to the effects of peer victimization on children’s adjustment and found that techniques such as critical problem solving were advantageous for non-victimized children, but tended to exacerbate troubles for victimized adolescents [10]. Correspondingly, a study that measured the effects of exposure to violence on problem behavior (adjustment outcomes) among 306 African-American middle and high school students found a linkage between victimization (including peer victimization) and avoidance as a coping strategy [11]. With reference to specific gender differences, the findings of this research demonstrated a stronger impact of victimization on offenses, self-rejection, and avoidance among young men and a greater impact of victimization on depression among young women in the select sample. Also, from the research, there is support to suggest that prevention and intervention strategies that are reliant on strengthening protective factors would be more operative in decreasing risk factors by bearing in mind variations not only in types of victimization but in adjustment outcomes as well. As an extension of this study, the researchers later examined parental background and traumatic victimization by family and peers as risk factors for violent delinquency among 208 urban adolescent females between the ages of 14–16. When investigating traumatic and repeated victimization, 14% had been assaulted, 15% had been approached and threatened with a gun, 12% had been assaulted sexually, 38% had witnessed someone being killed or badly hurt, and 62% reported being anxious and upset after viewing a dead body resulting from gun violence. Hence, the results of this study highlighted the strong connection between familial background, traumatic and repeated victimization, and violence and how these factors affect adjustment outcomes among African-American females in particular. By developing and implementing more after-school and community-based programs that specifically target African-American female adolescents; the authors posited that more alternatives would lead to reduced delinquent behavior and disproportionately lower crime rates [12].

Regarding specific coping approaches and violent victimization, including bullying, another study [13] focused on self-efficacy and variations in coping strategies among children. The research focused on numerous handling processes that have been recognized from the intervention literature, including problem resolving, pursuing social support, engagement resolution, reduced assertiveness, avoiding revenge-related behavior, avoiding internalizing behavior, avoiding externalizing behavior, and others. The study advanced a psychometrically comprehensive portion of children’s beliefs in their abilities to use their specific coping strategies. Likewise, additional research [14] examined the influence of social stress on indications of psychopathology at entrance into the adolescent stage. Overall, peer-related stress and early pubertal maturation were magnitudes of social stress that modeled specific challenges for the girls in this sample. More specifically, the discoveries addressed the use of cognitive behavioral therapy approaches by educators, therapists, and clinicians to help teens cope with potentially stressful experiences while curtailing symptoms of psychopathology. In addition, another study [15] inspected the association between children’s emotional reactions to peer victimization and their favored choice of coping approaches. Hence, outcomes indicated through bivariate associations that children who are victimized have more penetrating reactions to peer violence specifically. 

Additional research on gender differences has suggested variations in coping among boys and girls in response to victimization, including violence by peers. For example, one study [16] investigated the extent of two major forms of peer victimization, physical and interpersonal, and externalizing behaviors that included drug use, physical and relational aggression, and delinquency. While boys were more frequently exposed to physical victimization, relational victimization was relevant to both genders. The article focused on the risk factors that peer victimization creates with regard to aggression, delinquency, and drug use, and the results showed a stronger relation in physical victimization among boys, while interpersonal victimization experiences were related to high stages of physical and relational aggression among both boys and girls. Similarly, additional research [4] asserted that peer victimization among male and female adolescents signified a serious fear, given the incidence with which gun violence in particular occurs. Additional studies have examined the overall influence of peer victimization on internalizing and externalizing symptoms, including sleep inconsistencies and poor emotional regulation [17]. In one study, the results suggested that shorter sleep durations were directly linked to youths’ internalizing and externalizing symptoms, and had adverse effects on maladaptive responses [17]. Other studies have noted that age differences also exist regarding adolescents’ response to adverse environments as evidenced through internalizing and externalizing symptomatology. For example, research has found that older adolescents/youth (18+ year olds) are more likely to engage in delinquency as a response to victimization, while pre-adolescents (12–14 year olds) and middle adolescents (15–17 year olds) are more likely to exhibit internalizing symptoms such as anxiety and depression, much of which is in response to bullying and harassment by older youths [18]. Hence, studies continue to highlight the effects of age and gender on the development of symptoms indicative of violent victimization. Moreover, many researchers have followed the developmental psychopathology framework to conceptualize the risk and protective factors related to peer victimization, while concurrently stressing the multiple pathways that children chart and how features of victimization in a child’s life can change over time [19]. Here, they found that mother–child relationships and friendships are each connected with peer victimization, and the student–teacher relationship is associated with child social outcomes. Hence, studies such as these are particularly important in investigations of minority youth across dimensions of age and gender, since they may highlight family, community, and school patterns that may influence coping strategies as buffers to daily stressors, including increased rates of violent victimization, particularly when the peer group is considered [12].

Discoveries of the current study are based on the outcomes of broad research to approximate the level of violent victimization among urban youths. Numerous children and adolescents from disadvantaged upbringings have social, academic, and family involvements that not only disturb their development, but can also embolden delinquent behavior or criminal activity. We follow the aforementioned transactional relationship between stress and coping as our guiding theoretical framework in that it explains the relationship between coping mechanisms and delinquent behavior among minority adolescents. Thus, this investigation presents an addition to preceding work [4] that discusses the effects of violence on adjustment outcome, and delivers evidence on the influence of inner-city violence on behavior problems among urban youth using extended measures of exposure to violence, adjustment outcomes, and coping approaches. Stress is placed on the need for amplified intervention in the lives of youth at risk of community and school violence. Therefore, the study reports the resulting hypotheses based on survey data collected from 500 African-American adolescents:

- Socio-demographic factors will influence internalizing and externalizing consequences including defeatism, despair, worry, and delinquency. Younger adolescents will display more internalizing symptoms, while older youth will exhibit greater externalizing consequences. Additionally, males will exhibit higher rates of externalizing problems, while females will display more internalizing symptoms.
- Direct contact with violence, including involvement with peer victimization, will have an impact on internalizing and externalizing consequences including defeatism, despair, worry, and delinquency. Exposure to violent victimization will increase the occurrence of symptomatology among youth.
- Problem-focused/social support and emotion-focused/wishful thinking as coping strategies/protective factors will have an impact on internalizing and externalizing outcomes including defeatism, despair, worry, and delinquency. Problem-focused/social support will decrease the likelihood of symptomatology, while emotion-focused/wishful thinking will enhance the occurrence of symptomatology.
- There will be a moderating role of coping on victimization, including peer violence, with regard to internalizing and externalizing outcomes, including defeatism, despair, worry, and delinquency. Problem-focused/social support will decrease the positive effect of victimization on symptomatology, while emotion-focused/wishful thinking will increase the positive effect of victimization on symptomatology.

## 2. Research Design and Method

The methodology used in this paper is derived from reports of self-administered surveys completed by 500 adolescents between the ages of 12–18 in the state of Virginia. Census tract statistics were applied to obtain a stratified sample selected from various agencies, schools, churches, and community organizations that service youth in the Hampton Roads area of Virginia. In each occurrence, pupils who partook in the youth organizations and activities attended urban schools and had experienced gun-related violence and victimization (as targets, offenders, or witnesses) out of school. Moreover, all of the participants lived in zones that were categorized by modest to extraordinary violence in the Hampton Roads area of Virginia, as illustrated by police department figures. Parental salary, educational status, and work-related status served as measures of the adolescents’ socioeconomic context. Participants were recruited through the aforementioned community organizations and events, including health fairs, and by charting districts through flyers and door-to-door visits. Eligible respondents (i.e., those who lived in areas that were considered to have modest to extraordinary violence) were then scheduled for assessment completion, which was conducted at various sites, including houses of worship, schools, and community establishments. Approval was obtained from the Institutional Review Board, study #57033. Trained student research assistants collected the consent and assent forms, and 20–30 youths at a time were surveyed in small group layouts during group assemblies. Surveys lasted approximately 90 minutes, and participants received $10 for data collection both as an incentive and to minimize obstacles to participation. We met no problems with refusals to partake. However, it should be noted that the stratified sample is not generalizable, given the exact technique of recruitment and eligibility requirements. Descriptive statistics reveal that the sample was 89% Black, and fewer respondents were White, Hispanic, Asian, Native American, and Other. The greatest number of students was 15 years old and the modal grade was ninth. The majority (57%) of the students were male. The majority of the living arrangements included mother only (49%), followed by both parents (34%).

### 2.1. Exposure to Violence Measures

Participants finished portions of the Survey of Children’s Exposure to Community and School Violence [11], which processes level of exposure to both violence and victimization in the family, at school, and in the communal setting. Respondents were asked how often had they witnessed or experienced certain occasions at the start of the school year, including responses such as ”threatened with a gun”, “shot at with a gun”, “seen others attacked with a gun”, “seen others threatened with a gun”, “seen a dead body”, and “been to parties where guns were fired”. Objects were counted by field (communal, family, and school) to assess both straight and unintended exposure to violence, with higher marks indicating augmented exposure to violence. Higher marks specify greater relationship with violence, including both violence by peers and knowing other peers who have been either victimized or exposed to violence as victims or witnesses.

### 2.2. Internal and External Symptomatology Measures

The DSM (Diagnostic and Statistical Manual) Screener for Depression [20] investigates depression or despair among children and adolescents as they recollect feelings such as “been very sad”, “been so down that it is hard to do school work”, and “wished that I would die”, on a scale fluctuating from “hardly ever” to “almost every day”. A higher score signified severe depression. Fatalism or defeatism explores confidence in one’s ability to impact or not impact the future. Here, items were derived from a scale [21] in which participants replied to such statements as “people like me don’t have much of a chance in life” and “whether I get into trouble is just a matter of chance”. Answers alternated from strongly disagree to strongly agree, with higher scores representing increased fatalism or defeatism. Anxiety or worry was measured using sub-scaled questions from the Seattle Personality Questionnaire [22], which is a self-reported emotional symptomatology scale measuring current feelings such as “do you feel afraid a lot of the time”, do you worry about being teased”, and “do you have trouble paying attention in class”. Reactions were coded as no and yes, with higher scores representing increased anxiety. Concerning measures of self-worth, items were examined from Rosenberg’s Modified Self-Esteem Inventory [23], which inspects aptitude and self-respect with such statements as “I am convinced that I am a failure”, “I wish I could have more respect for myself”, and “I certainly feel useless”. Items were inverse scored, meaning that lower self-esteem was associated with better scores. Finally, delinquent behavior or wrongdoing was measured by self-report responses to three offense-type pointers [24], which includes a 14-item property crime index, a six-item violent crime index, and a three-item status offense index. For multivariate analyses with interaction relationships, items were further joined to make an internalizing behavior directory (anxiety and depressive symptoms, 60 items, alpha = 0.92) and an externalizing behavior directory (property-related and violent delinquency, 38 items, alpha = 0.83). To diminish difficulties with multicollinearity, the components of the interaction terms were standardized before multiplication. An examination of the difference inflation elements reveals that multicollinearity is not an issue. In particular, none of the inflation elements exceeds 4.00, which is the cutoff point that is normally recognized as a suggestion of multicollinearity complications [25].

### 2.3. Coping Strategies

Participants also finished a 54-item coping register [26]. Each item is rated on a five-point scale, demonstrating the occurrence with which a particular coping approach is used for managing specific difficulties. An orthogonal factor analysis was conducted by the primary author to produce 11 factors, including problem-solving coping, cognitive coping, adult social support, peer social support, parental support, substance use, physical exercise, aggression, social entertainment, individual relaxation, and prayer. Of specific prominence is Wills’ [26] use of predominately African-American adolescents for the inventory, addressing that the coping schemes used by these youths may vary from those used by others, given dissimilar life experiences. The 54-item coping portion has been shown to have high test–retest reliability, while correlations have provided support for the validity of the adolescents’ self-reports.

Factor loadings for the current project created two primary factors from the inventory: problem-focused/social support (positive ways of coping) and emotion-focused/avoidance (negative ways of coping). The first loading explained 37% of the variance, while the second loading explained 23% of the variance. Therefore, 60% of the total variance was explained by these two factors. In each instance, factors were saved as regression scores whereby increased scores denoted an increase in both positive ways of coping and negative ways of coping.

## 3. Analysis and Findings

To inspect the significance of exposure to violence, victimization, and coping stratagems for externalizing and internalizing complications, hierarchical multiple regression analyses were used. The youth’s age and gender were entered first into the model, succeeded by coping approach. In the third step, peer victimization was entered. Consistent with previous explorations of gender differences and problem behaviors, the results in Table 1 show that males are more likely to display externalizing symptoms such as delinquent behavior, although the results showed no support for females displaying internalizing symptoms such as anxiety and depression, which is inconsistent with previous studies. Older adolescents are also more likely to reveal externalizing symptoms. However, age has no influence on internalizing symptoms. When entered in the next steps, both types of coping schemes add a significant volume to the explained variance in delinquent behavior, although they are more likely to elucidate externalizing behaviors when associated with internalizing symptoms. For instance, problem-focused/social support coping meaningfully decreases delinquent behavior among adolescents, while emotion-focused/wishful thinking coping pointedly intensifies delinquent behavior among adolescents. Emotion-focused/wishful thinking coping also knowingly increases the impact of internalizing symptomatology (anxiety and depression) among the sample, yet problem-focused/social support coping has no consequence. Violent victimization involvements are considerable forecasters of both internalizing and externalizing problems, and consequences yield that these events are the best interpreters of symptomatology. In addition, violent victimization and emotion-focused/wishful thinking coping expressively projects the degree of experienced externalizing problems and internalizing problems. Among the coping stratagems, emotion-focused/wishful thinking coping has the largest beta weight for internalizing problems, while problem-focused/social support coping does not influence internalizing symptoms.

In summation, age, gender, victimization, and coping strategy were assessed in the present study, and explained a significant amount of variance in adolescent problem behavior. Regarding externalizing problems, the more significant factors are emotion-focused/wishful thinking coping and victimization, while victimization is a better interpreter of externalizing problem behaviors. As described earlier, a negative coping strategy is significantly connected to both internalizing behaviors and externalizing behaviors. The following set of examinations involved the potential moderating part of coping approaches. In each illustration, exposure to violence and victimization was the greatest predictor of problem behavior and internalizing symptoms among adolescents. The significant main effects of emotion-focused/wishful thinking coping on both internalizing symptoms and externalizing symptoms, and exposure to violence and victimization on both internalizing and externalizing symptoms designate that these issues play an autonomous (additive) role in adolescents’ complete regulation. Moreover, the significant interactions indicate a moderating influence of coping strategy on problem behavior, but not internalizing symptoms. That is, coping plan has a different impression on behavior of higher and lower levels of violent victimization, further signifying that an upsurge in problem-focused/social support coping decreases the positive result of violent victimization on externalizing problem behaviors, while an upsurge in emotion-focused/wishful thinking coping increases the positive result of victimization on externalizing problem behaviors. Formal follow-up analyses of significant interactions were subsequently conducted through simple slopes to determine the effect of coping strategy on externalizing symptomatology at high and low levels of victimization. The results indicated a greater impact of problem-focused coping on externalizing problems at high levels of victimization (beta = −0.083, sig. = 0.035) in comparison to low levels of victimization (beta = −0.046, sig. = 0.000). Additionally, the results suggested a stronger influence of emotion-focused coping on externalizing problems at low levels of victimization (beta = 0.297, sig. = 0.000) in comparison to high levels of victimization (beta = 0.078, sig. = 0.000).

## 4. Discussion and Conclusions

This study described the relationship between exposure to violence and victimization, coping strategy, and adjustment outcome among urban African-American youths. The results revealed that victimization experience, as an indicator of exposure to violent events and peer violence, was a criterion for both internalizing and externalizing symptomatology. A problem-focused/social support coping strategy was adversely related to externalizing problem behaviors such as delinquency. Whereas emotion-focused coping predicted greater problem behaviors and internalizing symptoms, problem-focused coping predicted fewer problems behaviors, and was unrelated to internalizing symptoms. Still, the discoveries show diminutive sustenance for the significance of a problem-focused/social support coping strategy as a predictor of internalizing symptoms such as depression and anxiety. The outcomes also propose that coping strategy has a moderating impact in that the complete effect of coping variables on (externalizing) problem behaviors varied by stages of victimization experience among assemblies of adolescents. Thus, the usage of problem-focused/social support coping lessens the positive influence of violent victimization on externalizing problem behaviors such as delinquency, while the usage of emotion-focused/wishful thinking coping upsurges the positive influence of violent victimization on externalizing problem behaviors such as delinquency. Accordingly, there is confirmation to recommend that prevention agendas aimed at solidifying coping approaches would be operative in decreasing the influence of exposure to violence and victimization as a risk feature by bearing in mind variations in coping strategies applied by adolescents. 

These effects are constant with preceding studies that have addressed victimization and victimization involving peers, in particular with regard to problems with adjustment among youths [27,28,29]. Another study, for example, found in a meta-analysis of longitudinal scholarships substantial relationships between peer victimization and succeeding variations in internalizing problems [2], while other researchers found that peer victimization is connected to adjustment difficulties in youth, including violent behavior [1]. Likewise, other scholars in their research found that peer victimization facilitates stress in youth by intruding on the development of effective managing while fostering maladaptive strain responses [3]. Similar to the findings of the current research study, others found differences in approach and avoidant responses with regard to peer victimization [6], while additional researchers [5] identified two-way connections between peer harassment and boys’ self-blame and girls’ wishful thinking with regard to the willingness to intervene in violent events. Hence, the present study finds support for the relationship between adjustment outcomes and coping experiences of lower income minority youths affected by peer victimization. In an attempt to comprehend the influence of negative ecological impacts on individual adolescents, it is recommended that more consideration be given to situationally pertinent factors (i.e., regulated inequality, exposure to community and school violence) as opposed to person-centered features (i.e., hostility, aptitude) in the determination to recognize the difficult circumstances that minority youth experience. One limitation of the current research is that the selection had a higher proportion of adolescents with truncated stages of socioeconomic rank compared to the collective populace, since the statistics have been derived from youths from high-risk regions for abuse and succeeding misconduct. There is a prerequisite at that point to observe youths’ involvements in and decisions about violence in the general populace. Within this framework, it is contended that better chances should be assumed to let minority youths construe their own involvements with peer victimization, lending additional provisions to explanatory weight in social science research. Finally, exposure to violent victimization, including violence among peers, remains a cautionary symbol for imminent violent offending among adolescents, and since the youth surveyed in this scholarship are at a greater risk than others for such persecution, procedures and agendas aimed at averting harassment may be effective if they are concentrated on these groups. While the mechanisms employed in this study comprised a sequence of inquiries involving individual identifications concerning peer-related, community-related and school-related violent events, adjustment outcomes, and coping strategies, impending research should persist in exploring the association between these dynamics as contributing to scholarship addressing violence with the African-American youth population.

## Figures and Tables

**Table 1:** Hierarchical Multiple Regression Analysis Calculating Adolescent Symptomatology Scores from Exposure to Violence and Victimization and Coping Approach as Predictors.

	Externalizing Indicators		Internalizing Indicators	
Level/Predictor Variable:	Beta	R^2^	Beta	R^2^
Socio-demographic Variables		0.063		0.010
Age of Respondent	0.229 ***		−0.063	
Sex of Respondent	−0.100 *		0.078	
*Coping Strategy Variables*		0.082		0.054
Problem-Focused Coping	−0.137 *		−0.003	
Emotion-Focused Coping	0.147 **		0.213 ***	
Violent Victimization Variable	0.590 ***	0.418	0.432 ***	0.235
Interaction Terms		0.457		0.239
Victimization Measure (x) Problem-Focused	−0.417 ***		0.048	
Victimization Measure (x) Emotion-Focused	0.350 ***		0.082	

* The association is statistically significant at the 0.05 level. ** The association is statistically significant at the 0.01 level. *** The association is statistically significant at the 0.001 level.
